# Effectiveness of Topical Cyclosporin-A 0.1% Compared to Combined Topical Cyclosporin-A 0.1% with Topical Sodium Hyaluronate on Interleukin-6 Levels in the Tears of Patients with Dry Eye Disease

**DOI:** 10.3390/vision7020031

**Published:** 2023-04-03

**Authors:** Desti Priani, Habibah S. Muhiddin, Junaedi Sirajuddin, Hasnah B. Eka, Burhanuddin Bahar, Agussalim Bukhari

**Affiliations:** 1 Department of Ophthalmology, Faculty of Medicine, Hasanuddin University, Makassar 90245, Indonesia; 2 Hasanuddin University Hospital, Makassar 90245, Indonesia; 3 Department of Public Health Medicine, Faculty of Medicine, Hasanuddin University, Makassar 90245, Indonesia; 4 Department of Clinical Nutrition, Faculty of Medicine, Hasanuddin University, Makassar 90245, Indonesia

**Keywords:** dry eye, interleukin-6, sodium-hyaluronic, cyclosporin-A

## Abstract

Introduction: Pro-inflammatory cytokines are important contributors to dry eye disease (DED). The cytokine interleukin (IL)-6 has become a therapeutic target in several DED drug studies. This randomized controlled trial aimed to determine the effectiveness of topical cyclosporin-A 0.1% compared to the combination of topical cyclosporin-A 0.1% and sodium hyaluronate in reducing tear IL-6 levels in DED patients. Methods: The participants were 20 patients, each with two eyes, who had moderate-to-severe DED. Before and after treatment, the clinical degree of DED was examined in each group, using ocular surface disease index (OSDI) scores, tear break-up time (TBUT), fluorescent tests, and Schirmer I tests. In addition, tear samples were taken to examine IL-6 levels through the ELISA method. The results were analyzed using the *t*-test, Wilcoxon test, and Mann–Whitney test. The correlation between tear IL-6 levels and the severity of DED was analyzed using the Spearman correlation test. Results: The study showed a significantly lower tear IL-6 level, OSDI score, and degree of ocular staining after either topical cyclosporin-A 0.1% or a combination of topical cyclosporin-A 0.1% and sodium hyaluronate (all values *p* < 0.05). Conclusions: The combination therapy was superior in reducing tear IL-6 levels. In addition, a correlation existed between tear IL-6 levels and the severity of DED based on the TBUT, although it was weak and not statistically significant.

## 1. Introduction

Dry eye disease (DED) is a common inflammatory disease of the eye surface that significantly affects the quality of life [1]. Recent studies have shown that inflammation plays an important role in its pathogenesis [2,3]. Pro-inflammatory cytokines have been known to significantly contribute to DED. This is evidenced by the presence of cytokines in the cornea (interleukin (IL)-1β and CXC chemokine ligand motif (CXCL) 10) and conjunctiva (IL-1β, CXCL10, IL-6, tumor necrosis factor-α (TNF-α), IL-12α, and IFN-γ) in DED patients through activation of the toll-like receptor 4 (TLR4) pathway [4].

The cytokine IL-6 plays an immunostimulatory role in which it can increase Th17 cell activation, leading to an escalating inflammatory response on the ocular surface [5]. Unlike other pro-inflammatory cytokines, which are only active at specific phases of the DED pathogenesis, IL-6 is involved in all essential stages of the disease process. This cytokine functions in the afferent arm stage of DED; ocular surface cells damaged by desiccating stress release it to activate antigen-presenting cells (APCs) [6]. These APCs also release IL-6 cytokines into the lymph nodes, stimulating naïve T cells to differentiate into Th 17 cells, which play a significant role in autoimmunity and the chronicity of DED [1,6]. This mechanism is strengthened by several research results showing that IL-6 levels increased along with increases in other pro-inflammatory cytokines such as IL-1, IL-2, IL-8, IL-10, IL-17A, and TNF-α [5,7]. Liu et al. [5] and Sun et al. [8] found positive correlations of IL-6 levels in the tears of DED patients with the ocular surface disease index (OSDI) score and corneal fluorescein staining, along with a negative correlation of these cytokine levels with the Schirmer test and tear break-up time (TBUT).

IL-6 has also become a therapeutic target in several DED drug studies. Several therapies, such as artemisinin analogs, Visomitin (SkQ1), thrombospondin-1, mesenchymal stem cell therapy, and tofacitinib, function by modulating IL-6 in DED [9]. Cyclosporin-A has also been used as an effective anti-inflammatory and anti-apoptotic in DED [10,11,12]. Sodium hyaluronate has emerged as an option for artificial tears, resulting in subjective and objective improvements in DED [13]. However, until now, no study has compared the effectiveness of topical cyclosporin-A 0.1% to that of topical cyclosporin-A 0.1% combined with topical sodium hyaluronate against more complete clinical parameters in DED patients. More relevant articles included in recent systematic reviews and meta-analyses have only compared the effectiveness of topical cyclosporin-A against or in combination with artificial tears [14]. Therefore, this study aimed to determine the effectiveness of topical cyclosporin-A 0.1% compared to the combination of topical cyclosporin-A 0.1% and sodium hyaluronate in reducing IL-6 levels in the tears of DED patients.

## 2. Materials and Methods

This randomized controlled trial was performed at Hasanuddin University Hospital. The population was all DED patients examined at our institution from January 2022 to April 2022. This research received permission from the Biomedical Research Ethics Commission on Humans, Faculty of Medicine, Hasanuddin University, with the number 11/UN4.6.4.5.31/PP36/2022. The sample size was calculated using the Harry King nomogram diagram for 16 people. The participants were divided into two groups: group A (topical cyclosporin-A 0.1%) and group B (combination of topical cyclosporin-A 0.1% with sodium hyaluronate).

The participant inclusion criteria were adult patients aged >18 years classified as having moderate-to-severe DED. Patients were excluded if they met any of the following criteria: (1) ocular surface diseases, such as corneal ulcers, episcleritis, scleritis, and uveitis; (2) history of ocular trauma; (3) inflammation unrelated to DED; (4) history of eye surgery or using systemic or topical cyclosporin-A, corticosteroids, prostaglandins, or NSAIDs; and (5) contact lens use.

### 2.1. Cyclosporin-A

Topical eye drops (Ikervis^®^, Meiji, Indonesia) 0.1% concentration (1 mg/mL) were used, one drop once daily at night in both eyes for a one-month follow-up period.

### 2.2. Combination of Cyclosporin-A and Sodium Hyaluronate

Topical eye drops (Ikervis^®^, Meiji, Indonesia) concentration of 0.1% (1 mg/mL) were used, with a dose of one drop once a day at night in both eyes. This was followed by topical sodium hyaluronate eye drops (Hyalub^®^, Cendo Pharmaceutical, Indonesia) concentration of 0.1% (1 mg/mL) at a dose of one drop every 4 h in both eyes for the one-month follow-up period.

### 2.3. Clinical Severity of DED

The patients had characteristic symptoms of dry eye. This was an OSDI score of >13, as well as one of the types of examination evidence supporting the diagnosis of DED: a TBUT of ≤10 s, fluorescein test of grade I, and Schirmer test 1 of <15 mm.

### 2.4. OSDI Score

Standard instruments for evaluating symptoms can easily be used to support the diagnosis of DED if the OSDI score is >13. The assessment parameters were 0–12 (normal), 13–22 (mild dry eye), 23–32 (moderate dry eye), and 33–100 (severe dry eye).

### 2.5. Schirmer Test 1

The quantity in the tear film (evaluation of the secretory function of the main lacrimal glands) was assessed using Schirmer–Whatman’s 41 strip paper for 5 min in the lateral third of the inferior fornix, without topical anesthesia, and seeing the amount of wetting, measured in millimeters (mm). This examination was classified as normal if the wetting length was 15 mm, mild dry eye if the wetting length was 9–14 mm, moderate dry eye if the wetting length was 4–8 mm, and severe dry eye if the wetting length was <4 mm.

### 2.6. TBUT

The stability of the tear film was assessed by calculating the time between a complete blink and the appearance of the first dry spot on the cornea. The interpretation was >10 s (normal), 7–10 s (mild dry eye), 4–6 s (moderate dry eye), and ≤3 s (severe dry eye).

### 2.7. Fluorescein Test

This test uses a fluorescein strip to obtain sufficient staining of the conjunctival and corneal surfaces with epithelial damage. The test results indicate DED with >5 corneal spots, >9 conjunctival spots, or eyelid margin staining with a length of ≥2 mm and a width of ≥25%. The gradation of symptom severity is divided based on the Oxford pattern [15]: grades 0–1 (normal), grades I–II (mild), grades II–III (moderate), and grades IV–V (severe).

### 2.8. Clinical Examination Procedure

Clinical examinations were always performed by the same two clinicians, each of whom performed the procedure in the same order as Mrugacz et al. [16] and Cocho et al. [17].

### 2.9. IL-6 Cytokine Analysis of Tear Samples

The concentration of IL-6 from the tears of participants was measured using the enzyme-linked immunosorbent assay (ELISA) kit (catalog no. E0090Hu, Bioassay Technology Laboratory; Shanghai, China) according to the manufacturer’s protocol at Hasanuddin University Medical Research Center (HUMRC).

### 2.10. Statistical Analysis

All analyses were performed using SPSS version 17.0 (IBM Corp.; Armonk, NY, USA). Differences in mean IL-6 levels between groups were analyzed using the paired *t*-test method (to compare between the same treatment groups before and after treatment) and the unpaired *t*-test method (to compare between different treatment groups before and after treatment). If the results of the Shapiro–Wilk data normality test were not normally distributed, a non-parametric test was performed: the Wilcoxon or Mann–Whitney test. Differences in clinical severity of DED before and after treatment were also measured using these non-parametric statistics. Spearman’s correlation test was performed to assess the relationship between IL-6 levels in the tears and the severity of DED based on the TBUT value. A *p*-value of ≤0.05 was considered significant.

## 3. Results

This study followed 40 eyes of 20 patients with DED, but one participant was dropped out because he was lost to follow-ups. Accordingly, data were analyzed from 38 eyes of 19 patients. The characteristics of the participants can be seen in Table 1. No significant difference existed in the clinical parameters of DED assessment between the two treatment groups (all *p*-values > 0.05; Table 1).

A significant difference existed between the OSDI scores before and after treatment in the 0.1% topical cyclosporin-A group (*p* = 0.005) and the 0.1% topical cyclosporin-A combined with sodium hyaluronate group (*p* = 0.000). However, the reduction proportion was more significant in the combination group than in monotherapy (83.29% vs. 77.46%). No significant difference existed in the OSDI scores between the two groups before and after treatment (*p* = 0.307 and *p* = 0.586, respectively; Table 2). A significant difference was found between the TBUT values before and after treatment in the 0.1% topical cyclosporin-A group (*p* < 0.05) and the 0.1% topical cyclosporin-A combined with sodium hyaluronate group (*p* < 0.05 in both eye sides). However, the proportion of improvement was more significant in the combination group than in monotherapy (87.30% vs. 51.20%). No significant difference existed in TBUT scores between the two groups before and after treatment (*p* = 0.483 and *p* = 0.807, respectively; Table 2).

**Figure 1:**
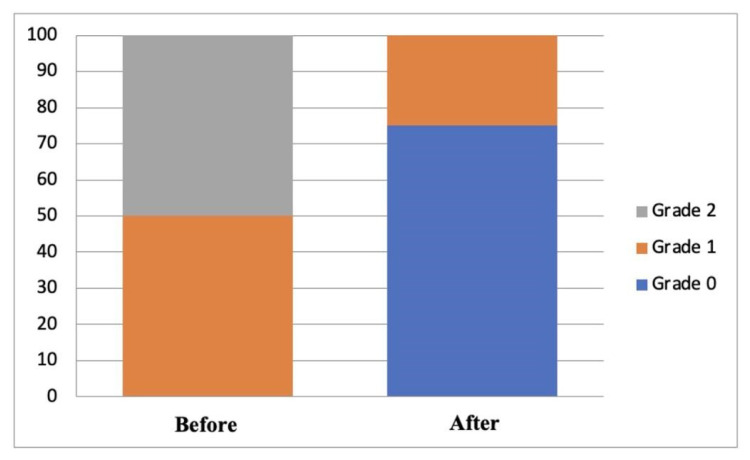
Distribution of degrees of ocular staining before and after treatment in group A.

**Figure 2:**
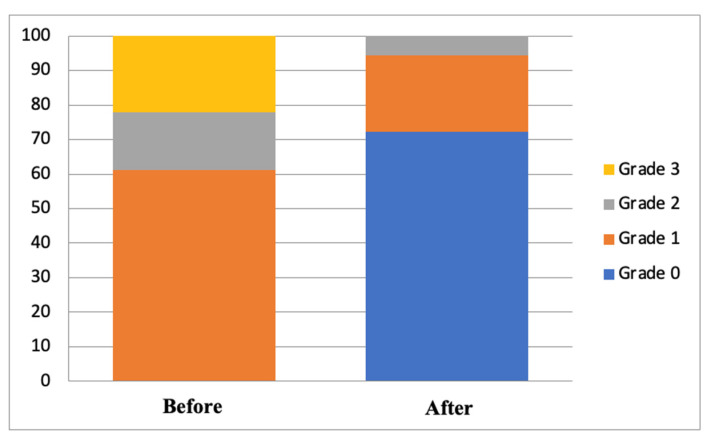
Distribution of degrees of ocular staining before and after treatment in group B.

A significant difference was found between the degree of ocular staining before and after treatment in the 0.1% topical cyclosporin-A group (*p* < 0.005) and the 0.1% topical cyclosporin-A combined with sodium hyaluronate group (*p* < 0.05). Both groups experienced an improvement in the degree of ocular staining, as seen from the shift in the severity proportion between before and after treatment, as shown in Figure 1 and Figure 2. However, no significant difference existed in the proportion of the degree of ocular staining between the two groups, either before or after treatment (*p* = 1.000 and *p* = 0.828, respectively; Table 3).

No significant difference was found between the Schirmer scores without anesthesia before and after treatment in the 0.1% topical cyclosporin-A group (*p* > 0.05) or the 0.1% topical cyclosporin-A combined with sodium hyaluronate group (*p* > 0.05). The Schirmer score without anesthesia in the monotherapy group increased by 33.30%. In contrast, the Schirmer score without anesthesia in the combination group decreased by 10.00%. However, no significant difference existed in the Schirmer scores without anesthesia between the two groups, before or after treatment (*p* = 0.671 and *p* = 0.657, respectively; Table 4).

A significant difference existed between the IL-6 levels in the tears before and after treatment in both groups (*p* < 0.05). Although IL-6 levels decreased after treatment in both groups, the most significant proportional decrease was observed in the combination group compared to the monotherapy group (24.85% vs. 15.66%). Table 4 also shows no significant difference in IL-6 levels in the tears between the two groups, before and after the treatment was given.

A comparison of the DED severity based on OSDI scores before and after treatment in the two groups can be seen in Table 5. A significant difference existed between the severity of DED before and after treatment in the 0.1% topical cyclosporin-A group (*p* = 0.004) and the topical cyclosporin-A 0.1% with sodium hyaluronate combination group (*p* = 0.004). However, no significant difference was found in the proportion of DED severity based on the OSDI score between the two groups, both before and after treatment (*p* = 0.292 and *p* = 0.081, respectively; Table 5). A significant difference was found between the severity of DED before and after treatment in the 0.1% topical cyclosporin-A group (*p* < 0.05) and the 0.1% topical cyclosporin-A with sodium hyaluronate combination group (*p* < 0.05). In addition, a significant difference existed in the proportion of DED severity based on the TBUT value between the two groups, both before and after treatment (*p* = 0.002 and *p* = 0.023, respectively). The mean rank of the combination group was consistently lower than the monotherapy group, both before treatment (14.61 vs. 23.90) and after treatment (15.58 vs. 23.02; Table 5).

**Figure 3:**
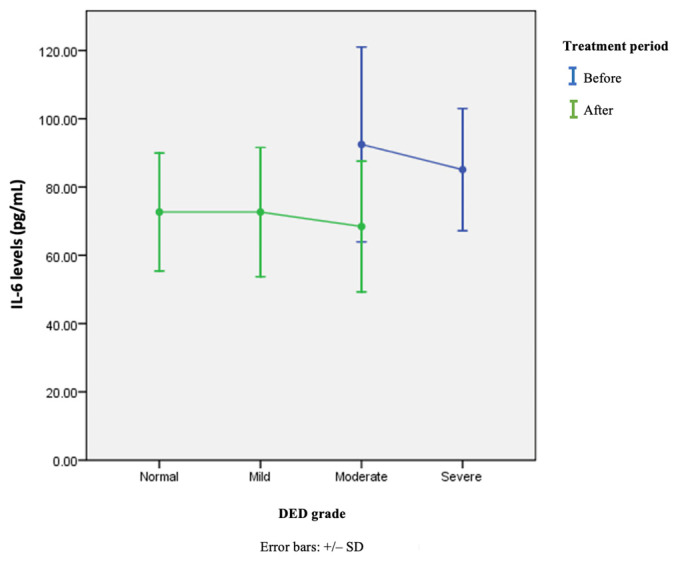
Average changes in IL-6 levels in tears at various degrees of DED severity based on TBUT values before and after treatment.

These results indicated a treatment effect on the improvement of DED clinical parameters, along with a decrease in IL-6 levels in the tears. No significant difference existed in the average IL-6 level in the tears at various degrees of DED severity based on TBUT values before or after treatment or with both combined (*p* = 0.391, 0.819, and 0.198, respectively; Table 6). However, the average value of IL-6 levels tended to increase with increasing DED severity, as shown in Figure 3.

The results in Table 7 show a positive correlation between IL-6 levels and OSDI scores, Schirmer test scores, and degree of ocular staining (ρ = 0.104, 0.156, and 0.239, respectively). These results indicate that the higher the level of IL-6 in tears, the higher the OSDI score, Schirmer test value, and degree of ocular staining in this study sample. Nonetheless, the correlations between IL-6 levels and these clinical parameters were very weak and not significant (OSDI: ρ = 0.104, *p* = 0.535; Schirmer: ρ = 0.156, *p* = 0.177) except for the ocular staining degree parameter, which had a weak significant correlation (ρ = 0.239, *p* = 0.038). Additionally, IL-6 was negatively correlated with the TBUT value; this was very weak and not significant (ρ = −0.132, *p* = 0.257). The higher the level of IL-6 in tears, the lower the TBUT value in this study sample. These results suggest that the higher the level of IL-6 in tears, the shorter the time between a complete blink and the appearance of the first dry spot on the cornea, so the severity of DED was more severe. This was reinforced by the results of the correlation test for the DED severity based on the TBUT value, which found a positive and significant correlation between these parameters and IL-6 levels. However, the correlation was still weak (ρ = 0.259, *p* = 0.024).

## 4. Discussion

In this study, the demographic data and initial clinical characteristics of the patients in the two groups were similar, and the treatments were randomized to avoid bias. However, a significant difference existed in the proportion of gender, with more women in the topical cyclosporin-A 0.1% with sodium hyaluronate combination therapy group. Several recent studies have shown that women have a higher prevalence and severity of DED than men [18,19], but this is not an independent factor [20]. In this study, the clinical characteristics of initial DED in the two groups were similar, so this could be considered not related to differences in gender balances of the two groups.

The DED clinical parameters of OSDI score, TBUT value, and degree of ocular staining experienced significant improvement in both treatment groups. This shows the effectiveness of the two ingredients in improving the clinical condition of DED, even though they have only been used for a relatively short time. Chen et al. [21] found similar results, where moderate–severe DED patients who received topical cyclosporin-A 0.05% experienced significant improvement in DED symptoms from day 28 and ocular surface test results on day 7 (all *p*-values < 0.05). Earlier studies also reported improvements in the first month of subjective symptom scores, Schirmer score, and TBUT in patients with moderate-to-severe DED who received topical cyclosporin-A 0.05%, either in combination with 1% methylprednisolone acetate or as monotherapy [11].

The proportion of improvement was consistently higher in the topical cyclosporin-A 0.1% with topical sodium hyaluronate combination therapy group compared to the group with topical cyclosporin-A 0.1% only. Previous studies found similar results: the combination of topical cyclosporin-A with sodium hyaluronate always showed superior efficacy in the clinical improvement of DED compared to cyclosporin-A monotherapy or topical sodium hyaluronate [22,23,24]. However, a recent multicenter study showed that 0.15% sodium hyaluronate was equivalently effective and safer than 0.05% cyclosporin-A in treating moderate-to-severe DED [25]. That study had a longer follow-up period than this study, but the doses used were different. The sodium hyaluronate and cyclosporin-A composition in the present study were the same (0.1%), so the drug dose could not determine the observed outcome differences. However, in the previous study, the sodium hyaluronate content was up to several times higher than the cyclosporin-A (0.15% vs. 0.05%). These discrepancies may achieve DED improvements similar to those of the two drugs.

The superiority of combined therapy in this study may be due to the synergistic effect obtained from sodium hyaluronate. This substance is widely used today as an artificial tear agent and has been shown to produce subjective and objective improvements in DED [13]. Sodium hyaluronate shows superior efficacy in several clinical parameters of DED compared to other artificial tears. A recent meta-analysis showed that the sodium hyaluronate group significantly increased tear production (based on the Schirmer test) compared to the non-sodium hyaluronate group (SMD 0.18; 95% CI 0.03, 0.33) with low heterogeneity (I^2^ = 0.0%, *p* = 0.632). However, no significant difference was found in the corneal fluorescein staining score, TBUT, or OSDI score in the sodium hyaluronate and non-sodium hyaluronate groups [26].

As a type of artificial tear, sodium hyaluronate has several mechanisms of action in repairing DED. First, it can inactivate the CD44 adhesion molecule, stabilizing the ocular surface barrier and the tear film, creating a favorable ocular surface microenvironment that enhances cell adhesion and motility and promotes cellular migration. Second, the high viscosity of sodium hyaluronate creates a retention effect of aqueous and secretory mucins in the tear film, thereby reducing friction between the cornea and eyelids during extraocular movements and blinking. This causes mechanical damage to the cornea, indirectly reducing the release of inflammatory cytokines and chemokines from the eye surface. Third, sodium hyaluronate has significant water resistance properties—with an affinity of 1000 times its weight. This increases the wettability of the ocular surface and reduces tear evaporation. Fourth, sodium hyaluronate has an anti-inflammatory effect that can maintain the expression of Muc5AC secretory mucin. This effect can also increase the stability of the tear film. Fifth, the sodium hyaluronate component can also rapidly react with fibronectin, stimulating the adhesion and extension of ocular epithelial cells and providing good support for controlling multiple DED symptoms. Regarding the IL-6 tear levels, sodium hyaluronate may hinder the abundant secretion to the ocular surface through its high viscosity to create a retention effect of aqueous and secretory mucins in the tear film. Further, lowering friction during blinking and extraocular movements will occur, lessening the release of inflammatory cytokines and chemokines from the surface of the eye due to mechanical damage to the cornea [13,27,28,29,30].

Cyclosporin-A was the primary agent to improve the DED outcome in this study. Cyclosporin has two mechanisms of action to this advantage. The first step is cyclosporin binding to cyclophilin, an intracellular receptor, and an immunophilin analog to interfere with molecular processes in T cells. This mechanism prevents T cells from being activated by APCs. The second step is binding the calcineurin complex and its inactivation. This step prevents dephosphorylation of the nuclear factor of activated T cells (NFAT) and its activation. As a result, IL-2 transcription is not initiated, and IL-2 is not released. Acting early in the cycle by inhibiting resting lymphocytes in the G0 and G1 stages of the cell cycle, cyclosporin causes programmed cell death (apoptosis) [31].

No significant changes were found in the parameters of the Schirmer test without anesthesia in the two treatment groups. These results differ from previous studies, which have generally shown that topical cyclosporin-A can significantly improve Schirmer test results [11,32]. Likewise, the combination of topical cyclosporin-A and sodium hyaluronate can increase the results of the Schirmer test [23,24]. This might be due to differences in the type (evaporated dry eye (EDE) vs. aqueous deficiency dry eye (ADDE)) and severity of DED [32]. The results of the Schirmer test did not improve in the efficacy study of cyclosporin-A on EDE patients [33]. The lipophilic nature of cyclosporin-A in various eye drop formulations may be the other factor that contributed to a different result in this study [34].

On the other hand, the Schirmer test results improved in ADDE studies [35,36]. Most participants in the present study belonged to the EDE type, where the average Schirmer score before treatment in the topical cyclosporin-A group was 13.00 ± 12.50 mm and the combination therapy group was 18.89 ± 10.82 mm. These insignificant results could also be since the duration of therapy in this study was short, so the maximum effect was not achieved. Furthermore, based on the severity of DED, the Schirmer test results improved in moderate-to-severe Sjögren syndrome patients with a dose of 2% topical cyclosporin-A [37]. In contrast, the results of the Schirmer test did not show significant improvement in more severe Sjögren syndrome patients, with no improvement in perceived subjective symptoms [38].

IL-6 levels in the tear decreased significantly in both the combination therapy and topical cyclosporin-A 0.1% monotherapy groups. However, the reduction effect of the combination therapy was superior to monotherapy. This may be related to the more significant improvement in DED clinical parameters in the combination therapy group compared to topical 0.1% cyclosporin-A alone. Byun et al. [11] showed that topical cyclosporin-A 0.05% in combination therapy with topical methylprednisolone or monotherapy could significantly reduce IL-6 and IL-8 levels in the tears, along with an improvement in symptom scores, Schirmer score, and TBUT in the first month. Likewise, Kang et al. [12] found that topical 0.05% cyclosporin-A, in the form of either nanoemulsion or conventional emulsion, could reduce IL-6 levels in the tears during 12 weeks of observation, along with improvements in TBUT, cornea and conjunctiva staining scores, and OSDI scores.

IL-6 levels in tears in this study showed an increase directly proportional to the severity of DED based on TBUT. These results were almost the same as those of Roda et al. [39]. In patients with DED, the IL-6 value obtained a mark of 98.82 pg/mL, but in the control group, the value was 12.04 pg/mL. The control group IL-6 value found by Roda et al. [39] was much lower than the IL-6 value obtained in our study. This may be because the normal samples we examined were normal TBUT results after treatment interventions in DED patients rather than those who had not previously been diagnosed with DED.

This study did not show a significant difference in IL-6 cytokine levels between DED severity degrees. Biologically, this may be due to the many pro-inflammatory cytokines that can play a role in the severity of DED besides IL-6. Zhao et al. [40] found that only IL-8 and TNF-α cytokines were correlated with DED clinical parameters in the form of OSDI, TBUT, and meibomian scores. In contrast, IL-6 and IFN-γ cytokines did not correlate with these clinical parameters, even though their values were significantly higher than the control group. Similar study results were obtained by Lee et al. [3], who found no significant differences in the clinical parameters of DED in the form of OSDI scores, Oxford scale, TBUT, and Schirmer scores between the Sjögren syndrome and non-Sjögren syndrome groups. These two groups had significant differences in IL-6 cytokine levels in their tear samples. In addition, the clinical symptoms of DED in Sjögren syndrome are more severe than in non-Sjögren syndrome [41,42]. Statistically, this insignificant difference may be due to the non-uniform number of samples in each group of DED severity degrees. Unlike many other studies, Lee et al. [3] focused on looking for associations between tear cytokine levels and clinical parameters of DED by comparing them across several DED classification groups (Sjögren syndrome vs. non-Sjögren syndrome DED vs. control; graft vs. host disease (GVHD) vs. non-GVHD vs. control). With a uniform number of samples, they found a significant difference in tear cytokine IL-6 levels between the compared DED classification groups. In addition, they found a correlation between IL-6 cytokine levels and other pro-inflammatory cytokines and the clinical parameters of the DED examined [5,8,43].

IL-6 levels in tears between various degrees of DED severity based on TBUT values were weakly correlated. Nonetheless, the level of IL-6 appears to tend to increase along with the increasing severity of DED, as also shown by the results of a negative correlation test. This differs from previous studies, which showed that tear IL-6 concentrations were only significantly correlated with Schirmer test results and tear osmolarity but not significantly associated with TBUT values and OSDI scores [44,45]. On the other hand, more studies have shown a strong correlation between tear IL-6 cytokine levels and the severity of DED on various clinical parameters, including TBUT values [5,43,46,47,48]. The combination of TGF-B and IL-6 promotes the initial differentiation of naïve CD4+ T cells into Th-17, which produces IL-17, which eventually infiltrates tissues and causes inflammation. Thus, IL-6 plays an important role in the pathogenesis of DED. Elevated levels of IL-6 in tears are the result of not an evaporative effect but overproduction [5]. The IL-6 level can be used as an indicator to determine the disease severity and evaluate the efficacy of anti-inflammatory drugs for DED [45]. Besides the intraocular IL-6 level, tear IL-6 level is higher in surface ocular diseases, such as DED, keratitis, and keratoconus. Thus, inhibiting local immune reactions during active inflammation may be significant in managing inflammatory ocular diseases [49].

This study corroborates previous research regarding the efficacy of topical cyclosporin-A, as either monotherapy or combination therapy with sodium hyaluronate, in improving clinical symptoms of DED. The effectiveness of a combination of topical cyclosporin-A with sodium hyaluronate, which was superior to topical cyclosporin-A alone, was successfully demonstrated in this study, related to a decrease in tear IL-6 levels. In addition, high tolerability is an advantage of the topical administration of cyclosporin-A. The short- and long-term adverse effects of topical cyclosporin-A are relatively mild. The most common is a burning sensation in the cornea when applying the drug to the eye. This is especially true with topical cyclosporin-A, which uses olive or corn oil as a vehicle [50,51,52,53,54].

The limitations of this study are its relatively small sample size, relatively short follow-up time, and evaluation of only one inflammatory cytokine. Multicenter research with a larger sample size and a relatively long follow-up time, examining other inflammatory cytokines that play a role in DED (such as IL-8 and TNF-α) by both mRNA and ELISA, is needed to confirm the results of this study. Whereas cyclosporin-A (Ikervis^®^) requires a prescription, sodium hyaluronate eye drops are widely available over the counter. The advantage of the combination therapy is more acceptable only if it shows significantly more improvements over the sodium hyaluronate eye drops alone in future studies, considering the cost-effectiveness and the potential adverse effects of cyclosporin-A.

## 5. Conclusions

IL-6 levels in the tears of DED patients were lower in both treatment groups. However, IL-6 levels were lower in the 0.1% topical cyclosporin-A with topical sodium hyaluronate combination therapy group compared to the group using topical 0.1% cyclosporin-A alone. A correlation was found between the IL-6 levels of the tears and the severity of DED based on the TBUT value. Physicians can consider combination therapy using topical cyclosporin-A 0.1% and topical sodium hyaluronate in patients with DED who are severe or require better clinical results quickly. Further research is needed with a multicenter study design and a longer follow-up time to assess the stability of the improvement in clinical parameters achieved and complications that may arise in the long term.

## Figures and Tables

**Table 1:** Participant characteristics.

Variable	Group A *n* (%)	Group B *n* (%)	*p*-Value
Age (years)	41.90 ± 12.02	33.00 ± 18.00 ^#^	0.539 *
Gender (%)			
Male	6 (60)	0 (0)	0.011 **
Female	4 (40)	9 (100)	
Comorbidities (%)			
Yes	1 (10)	1 (11.1)	1.000 **
No	9 (90)	8 (88.9)	
OSDI score	41.79 ± 7.13	50.26 ± 16.24	0.307 ***
TBUT value (seconds)	5.00 ± 2.00	4.33 ± 1.00	0.483 *
Ocular staining (n, %)			
Grade 1	10 (50)	11 (61.1)	1.000 **
Grade 2	10 (50)	3 (16.7)	
Grade 3	-	4 (22.2)	
Schirmer values without anesthesia (mm)	13.00 ± 12.50	18.89 ± 10.82	0.671 *

Note: Numeric data with normal distribution are expressed as mean ± SD; ^#^ Numeric data with non-normal distribution are expressed as median ± interquartile range; * Mann–Whitney test; ** chi-square test; *** independent *t*-test; SD, standard deviation.

**Table 2:** Comparison of OSDI and TBUT scores in the two groups before and after treatment.

Group	OSDI Score (Mean ± SD)		Percentage of Decrease (%)	*p*-Value
	Before	After		
A	41.79 ± 7.13 ^$^	9.42 ± 4.37	77.46	0.005 *
B	50.26 ± 16.24	8.40 ± 2.84	83.29	0.000 **
*p*-value	0.307 ^#^	0.586 ^##^		
Group	TBUT score (mean ± SD)		Percentage of increase (%)	*p*-value
	Before (second)	After (second)		
A	5.00 ± 2.00 ^$^	7.56 ± 2.79	51.20	0.000 *
B	4.33 ± 1.00	8.11 ± 2.42	87.30	0.000 **
*p*-value	0.483 ^#^	0.807 ^##^		

Note: ^$^ Data not normally distributed, median ± interquartile range; * Wilcoxon test for comparison of the before and after results in group A; ** paired *t*-test for comparison of the before and after results in group B; ^#^ Mann–Whitney test for comparison of the group A and group B results before treatment; ^##^ independent *t*-test for comparison of the group A and group B results after treatment; SD, standard deviation.

**Table 3:** Comparison of the degree of ocular staining before and after treatment.

Group	Degree of Ocular Staining (n, %)		*p*-Value *
	Before	After	
A			
Grade 0	-	15 (75)	0.000
Grade 1	10 (50)	5 (25)	
Grade 2	10 (50)	-	
Grade 3	-	-	
B			
Grade 0	-	13 (72.2)	0.000
Grade 1	11 (61.1)	4 (22.2)	
Grade 2	3 (16.7)	1 (5.6)	
Grade 3	4 (22.2)	-	
*p*-value ^#^	1.000	0.828	

Note: * Wilcoxon test for comparison of the before and after results in group A and group B; ^#^ Mann–Whitney test for comparison of the group A and group B results before and after treatment.

**Table 4:** Comparison of Schirmer scores without anesthesia and IL-6 levels before and after treatment.

Group	Schirmer Scores without Anesthesia (Mean ± SD)		Percentage of Change (%)	*p*-Value
	Before (mm)	After (mm)		
A	13.00 ±12.50 ^$^	17.33 ± 8.07	33.30	0.589 *
B	18.89 ± 10.82	17.00 ± 4.24	−10.00	0.740 **
*p*-value	0.671 ^#^	0.657 ^##^		
Group	IL-6 levels (mean ± SD)		Percentage of decrease (%)	*p*-value **
	Before (pg/mL)	After (pg/mL)		
A	82.64 ± 19.96	69.71 ± 21.72	15.66	0.034
B	97.65 ± 28.35	73.38 ± 13.97	24.85	0.002
*p*-value ^##^	0.065	0.545		

Note: ^$^ Data not normally distributed, median ± interquartile range; * Wilcoxon test for comparison of the before and after results in group A; ** paired *t*-test for comparison of the before and after results in group B; ^#^ Mann–Whitney test for comparison of the group A and group B results before treatment; ^##^ independent *t*-test for comparison of the group A and group B results after treatment; SD, standard deviation.

**Table 5:** Comparison of the severity of DED based on OSDI scores and TBUT values before and after treatment.

Group	OSDI Scores (*n*, %)		*p*-Value *
	Before	After	
A			
Normal	-	7 (70)	0.004
Mild	-	3 (30)	
Moderate	-	-	
Severe	10 (100)	-	
B			
Normal	-	9 (100)	0.004
Mild	-	-	
Moderate	1 (11.1)	-	
Severe	8 (88.9)	-	
*p*-value ^#^	0.292	0.081	
Group	TBUT values (*n*, %)		*p*-value *
	Before (second)	After (second)	
A			
Normal	-	1 (5)	0.000
Mild	-	11 (55)	
Moderate	8 (40)	8 (40)	
Severe	12 (60)	-	
B			
Normal	-	6 (33.3)	0.000
Mild	-	9 (50)	
Moderate	16 (88.9)	3 (16.7)	
Severe	2 (11.1)	-	
*p*-value ^#^	0.002	0.023	

Note: * Wilcoxon test for comparison of the before and after results in groups A and B; ^#^ Mann–Whitney test for comparison of the group A and group B results before and after treatment.

**Table 6:** IL-6 levels in tears at various degrees of DED severity based on TBUT values.

Variable	IL-6 Levels in pg/mL (Mean ± SD)	*p*-Value
Before		
Normal (*n* = 0)	0	0.391 *
Mild (*n* = 0)	0	
Moderate (*n* = 24)	92.46 ± 28.54	
Severe (*n* = 14)	85.10 ± 17.88	
After		
Normal (*n* = 7)	72.68 ± 17.28	0.819 **
Mild (*n* = 20)	72.67 ± 18.92	
Moderate (*n* = 11)	68.44 ± 18.31	
Severe (*n* = 0)	0	
Combined before and after treatment		
Normal (*n* = 7)	72.68 ± 17.28	0.198 **
Mild (*n* = 20)	72.67 ± 18.92	
Moderate (*n* = 35)	84.91 ± 28.05	
Severe (*n* = 14)	85.10 ± 17.88	

Note: * independent *t*-test for comparison of the two-group data; ** one-way ANOVA test for comparison of more than two-group data.

**Table 7:** Correlation between IL6 levels in tears and DED severity based on OSDI Scores, TBUT value, Schirmer Scores, and ocular staining.

	IL-6 (pg/mL)			
Variable	Immediate Measurement Results		Degree of Severity	
	*p*-Value	*p*-Value	*p*-Value	*p*-Value
OSDI scores	0.104	0.535	0.305	0.062
TBUT value	−0.132	0.257	0.259	0.024
Schirmer Scores	0.156	0.177	−0.098	0.400
Ocular staining	0.239	0.038	0.239	0.038

Note: Spearman correlation test.

## Data Availability

The datasets generated during and/or analyzed during the current study are available from the corresponding author on reasonable request.
